# Can Shahindezh be an age-friendly city? a case study 

**Published:** 2019-08

**Authors:** Farzad Maleki

**Affiliations:** ^*a*^Department of Epidemiology and Biostatistics, School of Public Health, Tehran University of Medical Sciences, Tehran, Iran.

## Abstract

**Background:**

Population aging is one of the challenges of the 21st century in the world, and in Iran. Elderly people should be able to participate in social activities befitting their age and be respected. In the last ten years, following the efforts of the WHO to develop the age-friendly Program, the age-friendly community s movements have attracted the attention of policymakers. So, this study was carried out with the aim to investigate the comparison of Shahindezh city with the standard indices of Elderly-friendly City.

**Methods:**

This descriptive cross-sectional study was implemented in 100 elders (above 60 years) referring to health centers of Shahindezh city, Nov-Dec 2018. Samples were selected through multi-stage sampling from 3 health centers of the city. Inclusion criteria were age over 60 years and living in Shahindezh. The WHO standard questionnaire was used; its validity has been confirmed. SPSS software was used to analyze the data and T-test was used to compare the mean of the indices.

**Results:**

A total of 100 elderly people participated in this study. Their average age was 72.41 ± 9.81, with the most (35%) at 65 years old. 46% of them were male. The results show that among the social, communicational, cultural-health and health indices, the highest percentage of dissatisfaction was related to the lack of computer and Internet educational facilities for the elderly, the inadequacy of urban furniture for the elderly. Also, the necessary training is not given to families for the care of the elderly. Findings also show that the average of all the criteria (social, communicative, cultural-psychological, and health) is lower than the standards.

**Conclusions:**

It is suggested that policymakers plan to increase the indices with the least average compared to the standard and thus provide welfare and health to the elderly.

**Keywords:**

Age-friendly cities, Aging, Shahindezh, Iran

